# The role of epithelial–mesenchymal transition-regulating transcription factors in anti-cancer drug resistance

**DOI:** 10.1007/s12272-021-01321-x

**Published:** 2021-03-25

**Authors:** Jihye Seo, Jain Ha, Eunjeong Kang, Sayeon Cho

**Affiliations:** Laboratory of Molecular and Pharmacological Cell Biology, College of Pharmacy, Chung-Ang University, Seoul, 06974 Republic of Korea

**Keywords:** Drug resistance, Epithelial-mesenchymal transition, Transcription factor, Snail, Twist1, ZEB1

## Abstract

The complex orchestration of gene expression that mediates the transition of epithelial cells into mesenchymal cells is implicated in cancer development and metastasis. As the primary regulator of the process, epithelial-mesenchymal transition-regulating transcription factors (EMT-TFs) play key roles in metastasis. They are also highlighted in recent preclinical studies on resistance to cancer therapy. This review describes the role of three main EMT-TFs, including Snail, Twist1, and zinc-finger E homeobox-binding 1 (ZEB1), relating to drug resistance and current possible approaches for future challenges targeting EMT-TFs.

## Introduction

Metastatic cancer is the leading cause of cancer deaths worldwide, despite large-scale clinical trials by researchers to overcome it. The 5-year relative survival rate of lung cancer patients is 5%, with more than half of the patients diagnosed with metastatic disease (Howlader et al. 2015; Siegel et al. 2020). In addition, the 5-year survival rate after resection of metastatic ovarian tumors is less than 20% (Yada-Hashimoto et al. 2003). These cases emphasize that cancer metastasis plays a vital role in promoting cancer progression and reducing patient survival (Steeg 2006; Yeung et al. 2015).

**Fig. 1:**
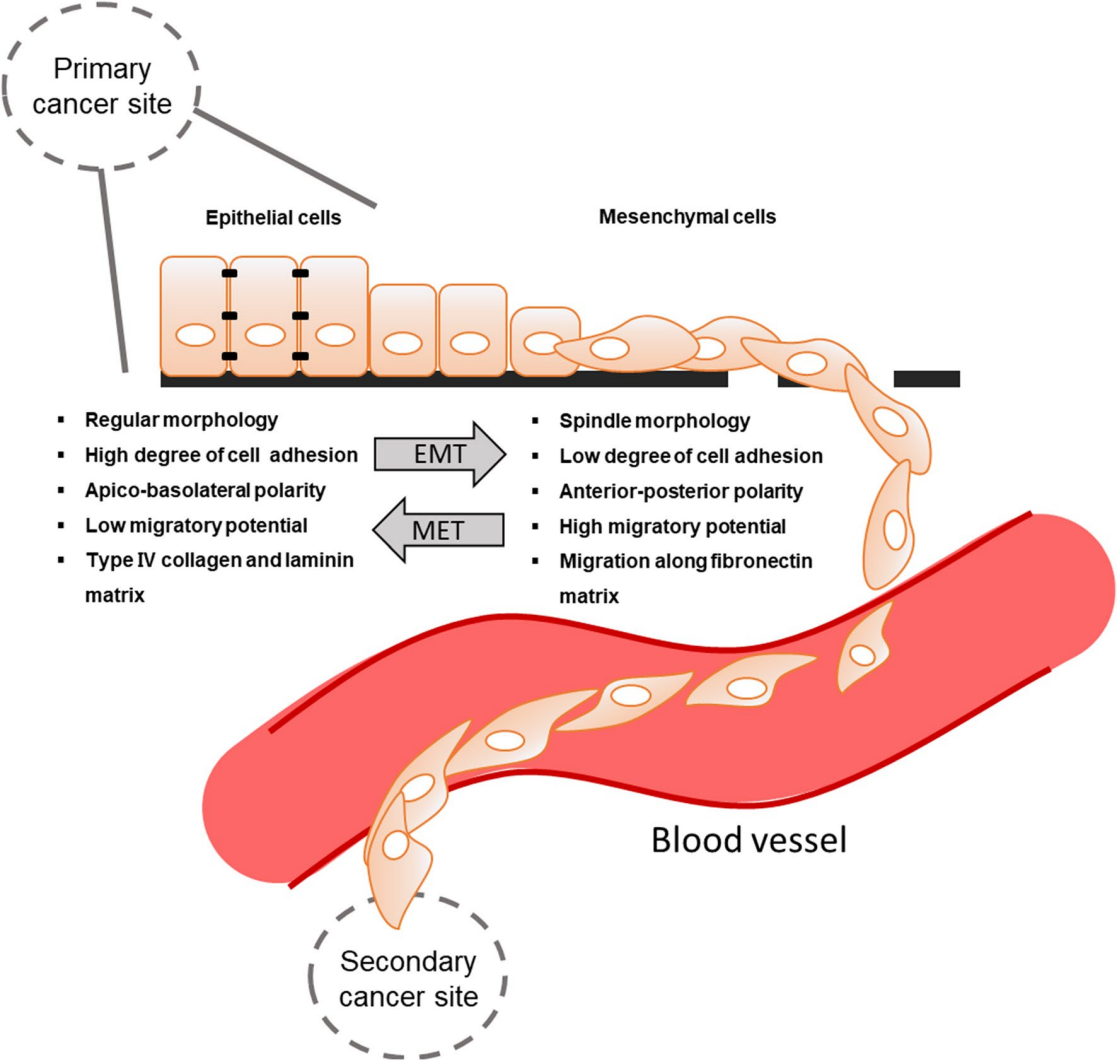
Epithelial–mesenchymal transition (EMT) in cancer metastasis. In the process of cancer metastasis, the characteristics of epithelial cells change to typical mesenchymal cells. These cells have the flexibility to penetrate blood vessels. They travel along the blood vessel and then settle to develop cancer. Cancer metastasis occurs when cells from the primary cancer site travel to a secondary site. MET, mesenchymal–epithelial transition

Cancer metastasis is caused by a reversible biological process, that induces the transition from non-metastatic cancer cells to metastatic cancer cells. In epithelial cancer, epithelial cells are converted into mesenchymal cells that have increased invasive properties due to loss of intercellular adhesion and thus gain of motility. The process is known as epithelial**–**mesenchymal transition (EMT), initially called epithelial**–**mesenchymal transformation (Fig. 1) (Kalluri and Neilson 2003; Kalluri and Weinberg 2009; Thiery et al. 2009; Bradley et al. 2013; Cho et al. 2019). EMT is pivotal in embryogenesis and is recognized as an essential process in cancer metastasis (Thiery 2003; Barrallo-Gimeno and Nieto 2005). Epithelial cells are characterized by apical**–**basal polarity, which helps cells tightly position each other on a basement membrane via intercellular junctions (Lamouille et al. 2014). Through EMT, cancer cells lose the cell**–**cell adhesion and gain enhanced motility and invasiveness. Several molecular processes need to be engaged during EMT initiation, such as the activation of transcription factors, expression of specific cell surface proteins and cytoskeletal proteins, production of extracellular matrix (ECM)-degrading enzymes, and changes in expression of a particular pool of microRNAs (Serrano-Gomez et al. 2016). In addition, cells that have undergone EMT often become resistant to apoptosis and senescence (Thiery et al. 2009).

Chemotherapy is the most widely used among various approaches to treat cancer (DeVita and Chu 2008; Min and Lee 2021). Unfortunately, drug therapies have become increasingly problematic because of resistant mechanisms in response to cancer-targeting drugs (Song and Faber 2019). Two types of drug resistance are recognized, namely intrinsic and acquired drug resistance. Intrinsic drug resistance exists in cancer cells before drug treatment, whereas acquired drug resistance develops after the exposure of cancer cells to drug treatment (Lippert et al. 2011). When multi-drug resistance occurs in cancer cells, the possibility of cancer metastasis and recurrence increases, and the clinical outcomes become worse (Hao et al. 2010). In addition, chemotherapy resistance has been observed with an increased ability to invade cells (Kajiyama et al. 2007; Işeri et al. 2011; Zhang et al. 2014). According to a recent study, the development of drug resistance leads to EMT through upregulation of EMT-promoting transcription factors in breast cancer (Mallini et al. 2014; Duran et al. 2017). Therefore, EMT has been investigated to understand the mechanism of cancer metastasis and responses to anti-cancer drugs (Oliveras-Ferraros et al. 2012; Wilson et al. 2014; Du and Shim 2016).

### EMT signaling and EMT-transcription factors

**Fig. 2:**
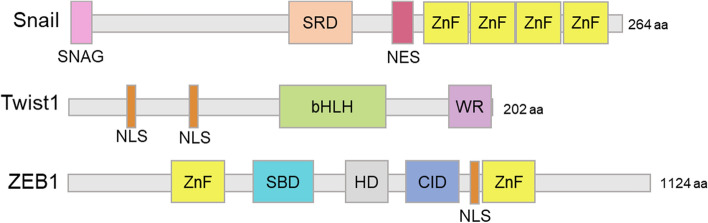
Structure of EMT-related transcription factors (EMT-TFs). The size scale of each domain reflects the domain sequence length within the amino acid sequence in each protein. The N-terminal is located on the left and C-terminal is located on the right. *SRD* serine-rich domain, *NES* nuclear export signal, *ZnF* zinc fingers, *NLS* nuclear localization sequences, *bHLH* basic helix-loop-helix, *WR* tryptophan and arginine motif, *SBD* Smad binding Dodoma, *HD* homeodomain, *CID* CtBP interaction domain

The gene expression pattern during EMT is regulated indirectly or directly by EMT-transcription factors (EMT-TFs) such as Snail, Twist1, and the zinc-finger E homeobox-binding 1 (ZEB1) (Fig. 2) (Bradley et al. 2013). Subsequent EMT induction by EMT-TFs is associated with invasion, propagation, metastasis, and cancer stem cell phenotype (Thiery et al. 2009; Puisieux et al. 2014). EMT-TFs are also correlated with resistance to chemotherapy, radiation, and targeted therapy (Sequist et al. 2011; Davis et al. 2014; Ansieau et al. 2014).

**Fig. 3:**
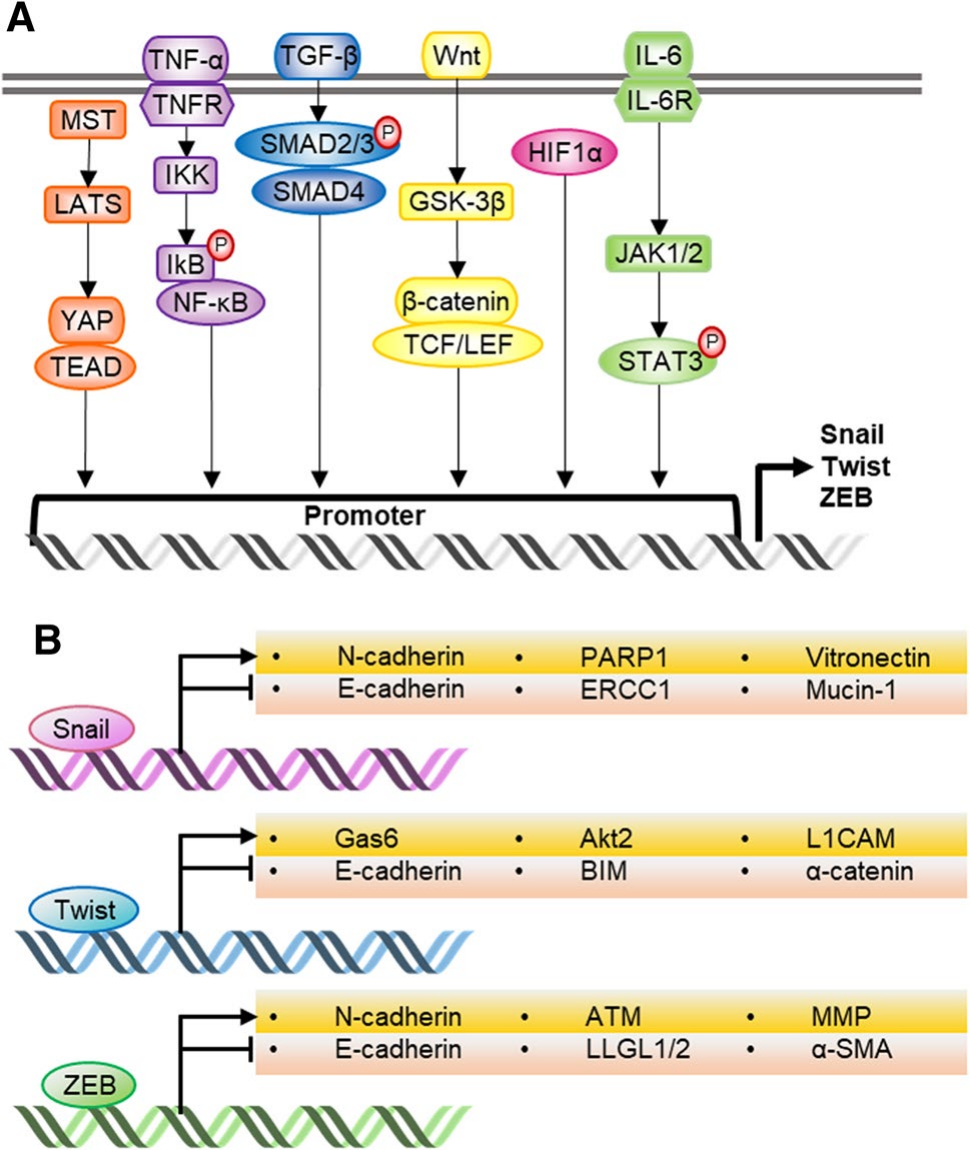
Key intracellular pathways and transcriptional target genes of EMT-TFs. **a** Several intracellular pathways induce transcription of EMT-TFs by binding to their promoter regions. **b** EMT-TFs regulate the expression of essential genes for EMT and drug-resistant related genes. *NF-κB* nuclear factor-κB, *IKK* IκB kinase, *TNF-α* tumor necrosis factor- α, *TNFR* tumor necrosis factor receptor, *TNF-β* transforming growth factor beta, *GSK-3β* glycogen synthase kinase-3β, *TCF/LEF* T cell factor/lymphoid enhancer factor, *HIF1α* hypoxia-inducible factor 1 α, *IL-6* interleukin-6, *JAK1/2* Janus kinase 1/2, *STAT3* signal transducers and activators of transcription 3, *PARP1* poly (ADP-ribose) polymerase, *ERCC1* excision repair cross-complementing group 1, *GAS6* growth arrest-specific 6, *L1CAM* L1 cell adhesion molecule, *BIM* Bcl-2-like protein 11, *ZO-1* zonula occludens-1, *ATM* ataxia-telangiectasia mutated, *LLGL1*/*2* lethal giant larvae protein homolog 1/2, *α-SMA* α-smooth muscle actin

EMT-TFs are regulated by various intracellular signaling pathways (Fig. 3a). The extracellular signal molecules bind to each specific membrane receptor and trigger intracellular signal transduction; growth factors, transforming growth factor beta (TGF-β), Wnt, and the Jagged family bind to receptor tyrosine kinase, TGF-β receptor, Frizzled, and Notch, respectively (Polyak and Weinberg 2009; Bradley et al. 2013; Lu and Kang 2019). Ligand-bound receptors transduce intracellular signals via the pathways, such as mitogen-activated protein kinase, phosphatidylinositol 3‑kinase (PI3K)/protein kinase B (Akt), nuclear factor-κB, β-catenin, or the Smad signaling pathway, which regulate the expression and stability of EMT-TFs (Bradley et al. 2013). EMT-TFs suppress the expression of epithelial markers, such as E-cadherin, cytokeratins, and tight junction proteins, and induce the expression of mesenchymal markers, including N-cadherin, vimentin, fibronectin, α-smooth muscle actin, matrix metalloproteinases (MMPs), and lethal giant larvae protein homolog 1/2 (Fig. 3b) (Kalluri and Weinberg 2009; Chao et al. 2014; Lu and Kang 2019). Reduced E-cadherin is the most representative indicator of cancer stemness and cancer resistance to treatment (Mani et al. 2008; Gupta et al. 2009). This orchestration of intracellular pathways and gene expressions regarding EMT-TFs is essential for the EMT process and the cancer therapeutic resistance correlated with EMT.

In the following, we will explore how EMT-related signaling factors affect drug resistance. Epidermal growth factor receptor (EGFR) is overexpressed in human cancers, and its activation is required for TGF-β1-induced EMT (Grandis and Sok 2004; Li et al. 2015). Thomson et al. suggested that the sensitivity to inhibition of EGFR depends on the degree of EMT occurrence in EGFR-expressing human non-small cell lung cancer (NSCLC) xenograft (Thomson et al. 2005). In particular, when E-cadherin was expressed, it showed higher sensitivity to EGFR inhibition (Thomson et al. 2005). Furthermore, a decrease of E-cadherin and an increase of vimentin was observed in gefitinib-resistant A549 lung adenocarcinoma cells. These cells also revealed cross-resistance against other EGFR tyrosine kinase inhibitors (EGFR-TKI) such as erlotinib and ZD6478 (Rho et al. 2009). These studies imply that the EMT process is vital in determining sensitivity to EGFR and its inhibitors. Notably, the extent of the EMT process is essential for predicting responses to the receptor, the uppermost molecule of intracellular signaling vital for cancer development.

Akt2, which is closely related to Twist1 in the EMT process, has also been associated with cancer resistance. Akt2 is thought to be responsible for chemoresistance to docetaxel via protection of survivin in A2780 or MDA-MB-231 cells (Xing et al. 2008a, , b). Other studies suggest that growth factors induce cell growth and resistance to tamoxifen by regulating PI3K/Akt2 signaling (Sun et al. 2001). In addition, Akt2 is involved in resistance to several chemotherapies regulated by HER2 in MCF-7 cells (Zhang et al. 2011).

MMPs are essential proteolytic enzymes that promote the migration of cancer cells to adjacent tissues through ECM breakdown (Chen and Parks 2009). Invasive cancer cells have a high level of MMP-2/9 expression, and the expression level of MMP-2/9 in drug-resistant cancer cells is higher than that of drug-sensitive cancer cells (Wattanawongdon et al. 2015). A study on MMP-2/9 expression and drug resistance conducted with epirubicin revealed that the expression of MMP-2/9 has been significantly higher in epirubicin-resistant cancer cells than non-resistant cancer cells (Zhang et al. 2015).

As described above, cancer resistance and the EMT process are closely linked to each other. The signaling molecules involved in EMT mediate the resistance to drugs in various types of cancer. The effects of EMT-TFs on cancer drug resistance are not limited to the function of EMT-TFs but may occur by the regulation of related signaling factors involved. Thus, the close association between EMT-related signaling factors and drug resistance suggests that EMT-TFs may be correlated with drug resistance.

### Drug resistance: Snail

Snail (Snail1) and Slug (Snail2) are reported to be associated with the EMT process during development of cancer, whereas functions of Smug (Snail3) are not revealed clearly. The Snail family shares a common C-terminal domain and various N-terminal regions (Barrallo-Gimeno and Nieto 2009). Snail proteins have multiple serine and proline residues near the middle region (Sefton et al. 1998). The C-terminal region of Snail is constructed with a DNA-binding domain that consists of zinc fingers (ZnFs) recognizing a common E2-box type element (CAGGTG) (Grimes et al. 1996; Hemavathy et al. 2000; Barrallo-Gimeno and Nieto 2009). The expression of Snail is associated with chemo- and radio-resistance through reduction of apoptosis and an increase in cell stemness (Smith and Bhowmick 2016).

Activation of P-glycoprotein (P-gp) mediates drug efflux transport, which then associates with multi-drug resistance (MDR). This activation is involved in cancer progression and is found in Snail-overexpressing NSCLC cells (Tomono et al. 2017). Overexpression of Snail influences the expression of P-gp (Tomono et al. 2017). Snail overexpression also leads to breast cancer resistance protein-mediated the MDR in MCF-7 breast cancer cell line (da Fonseca et al. 2016). Snail overexpression induces doxorubicin resistance, which subsequently causes EMT, and cells undergoing EMT show MDR through enhancing P-gp expression (Li et al. 2011). The EMT program and the induction of Snail play an acceptable role in gaining resistance to doxorubicin (Li et al. 2009). Snail-mediated upregulation of Poly [ADP-Ribose] Polymerase 1 has also been reported to contribute to doxorubicin resistance in human MDA-MB-231 breast cancer cells (Mariano et al. 2015).

Cisplatin is one of the broad and effective anti-cancer drugs (Shen et al. 2012). Co-expression of Snail and excision repair cross-complementing group 1 (ERCC1) in head and neck squamous cell carcinoma (HNSCC) patients correlated with cisplatin resistance and poor prognosis (Hsu et al. 2010). Snail knockdown induced inhibition of ERCC1 expression and attenuation of cisplatin resistance (Hsu et al. 2010). All Snail members contain a conserved SNAG domain at the N-terminus (1–9 amino acids), which is essential to repress transcription of target genes (Grimes et al. 1996; Batlle et al. 2000; Hemavathy et al. 2000; Peinado et al. 2004; Molina-Ortiz et al. 2012). The SNAG domain of Snail is a crucial mediator of ERCC1 transcription, and Snail improves sensitivity to cisplatin through inhibition of ERCC1 in HNSCC cells overexpressing Snail protein (Hsu et al. 2010). In addition, the mesenchymal phenotype is reversed and decreases resistance to cisplatin by reducing the expression of Snail in cisplatin sensitive (A2780) and resistant (A2780CR) ovarian adenocarcinoma cell lines (Haslehurst et al. 2012). These studies suggest that EMT-related transcription factors may play an essential role in cisplatin resistance in cancer. When Snail is knocked-out in cisplatin-resistant A2780CR cells, the drug sensitivity of these cells is restored, followed by reversing EMT phenotypes (Haslehurst et al. 2012).

**Fig. 4:**
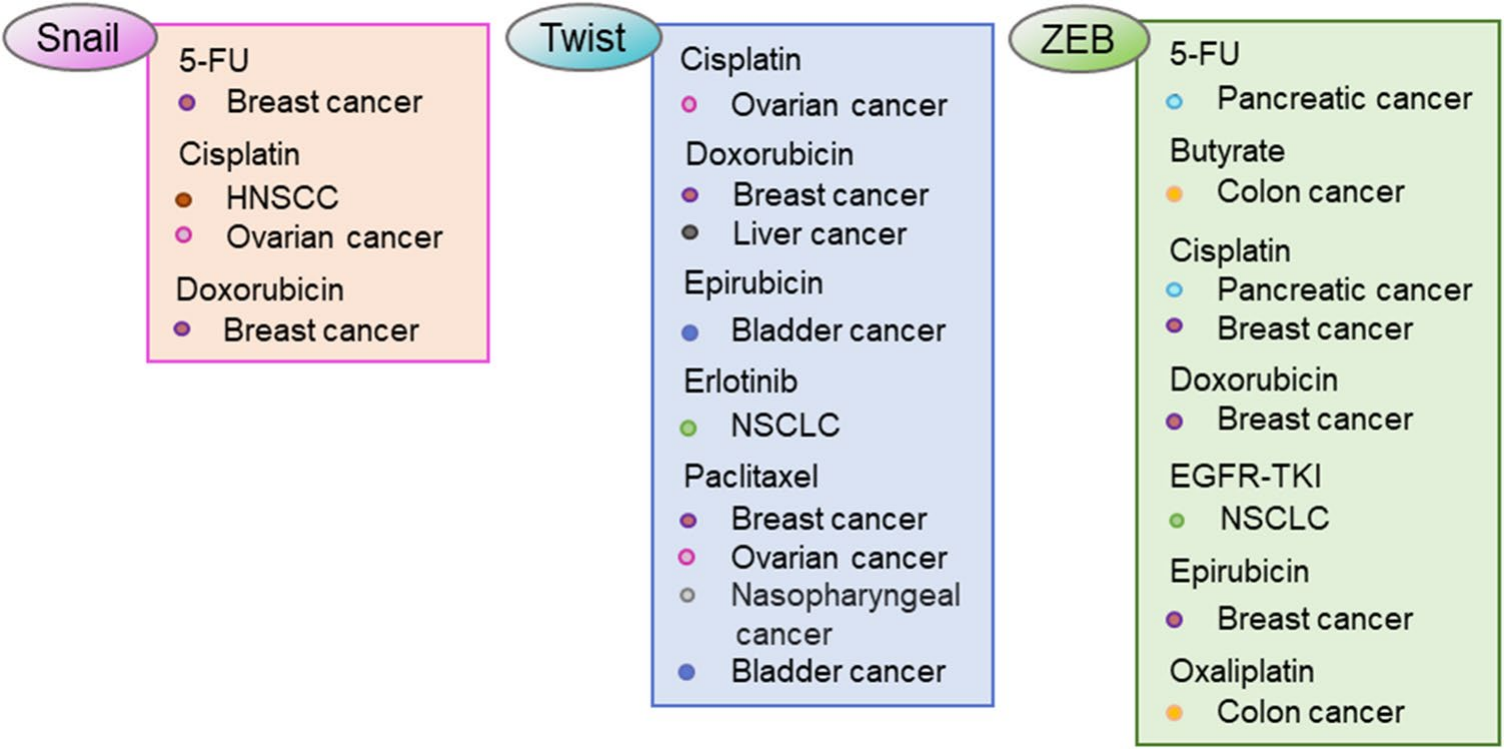
EMT-TFs-related resistant drugs and cancer types. The drugs are listed in relation to EMT-TF with the type of cancer for which drug resistance is reported. *HNSCC* head and neck squamous cell carcinoma, *NSCLC* non-small cell lung cancer

Snail is also involved in 5-fluorouracil (5-FU) resistance. When Snail is knocked-down in MCF-7 breast cancer cells resistant to 5-FU, cell invasion is decreased through reversing EMT, and sensitivity to 5-FU is improved (Zhang et al. 2012). Considering the numerous reports regarding Snail and the correlation with chemoresistance, targeting Snail to overcome cancer resistance is an attractive approach (Fig. 4).

### Drug resistance: Twist1

Twist1 was discovered originally in *Drosophila* as a basic helix-loop-helix family transcription factor (Simpson 1983; Thisse et al. 1987, 1988). Twist1 is reported to function as an essential transcription factor that regulates the EMT process, and eventually contributes to cancer metastasis. Twist1 is known to induce tumorigenesis in breast and prostate carcinomas, which is the result of EMT induction, invasion, and metastasis (Yang et al. 2004, 2008; Cheng et al. 2007, 2008; Pham et al. 2007; Ansieau et al. 2008; Zhuo et al. 2008). Twist1 expression induces a morphological change associated with EMT and significantly elevates cancer stem cell-like traits in MCF-7 and cervical cancer HeLa cells. Twist1 is also implicated in playing an essential role in the expansion of cancer stem cells and resistance to chemotherapy (Qin et al. 2012). Twist1 may be involved in other tumor-promoting effects, such as chemical resistance to metastasis and invasiveness associated with common chemotherapy (Li and Zhou 2011; Owens and Naylor 2013). Twist1 is not only involved in the development of acquired drug resistance in human cancer cells but is also associated with chemotherapy resistance when overexpressed, leading to a poorer prognosis (Lu et al. 2014; Deng et al. 2016; Liu et al. 2017). Interestingly, β-catenin and Akt pathways are activated in Twist1-overexpressing cells (Li and Zhou 2011).

Twist1-overexpressing breast cancer cells up-regulated Akt2 transcription, which promotes resistance to paclitaxel (Cheng et al. 2007). Similarly, resistance to paclitaxel by Twist1 overexpression occurs in human nasopharyngeal carcinoma cells, bladder, and ovarian cancer cells (Wang et al. 2004).

Erlotinib is an ATP-competitive tyrosine kinase inhibitor specific to EGFR, which is approved by the Food and Drug Administration for targeting NSCLC (Kosaka et al. 2011). Twist1 overexpression causes resistance to erlotinib in NSCLC models both in vitro and in vivo (Yochum et al. 2019). The erlotinib resistance mediated by Twist1 is due partially to the suppression of transcription of *BCL2L11*, a pro-apoptotic gene, by Twist1 (Yochum et al. 2019). Inhibiting Twist1 by genetic silencing or a chemical inhibitor is sufficient to overcome resistance to erlotinib (Yochum et al. 2019).

Twist1 is also suggested to be a useful biomarker for predicting resistance to doxorubicin (Li et al. 2009), used widely for adjuvant chemotherapy of breast cancer (Demir et al. 2019). *TWIST1* expression level is higher in the doxorubicin-resistant samples than the doxorubicin-sensitive samples collected from breast cancer patients (Demir et al. 2019). When Twist1 is overexpressed in cisplatin and doxorubicin-resistant cancer cells, it modulates Y-box-binding protein-1 as a downstream target (Shiota et al. 2011). This correlation increases cell growth, invasion, and drug resistance (Shiota et al. 2011). Interestingly, silencing *TWIST1* restores doxorubicin sensitivity in HepG2 liver cancer cells (Li et al. 2018). The positive correlation of Twist1 and multi-drug resistance protein 1 (MDR1) is observed in cancerous liver tissues, which are associated with EMT markers (Li et al. 2018). The down-regulation of Twist1 leads cancer cells to be sensitive to doxorubicin by suppression of MDR1 and EMT (Li et al. 2018). Chemotherapy increases the expression of Twist1 and several ATP-binding cassette transporters in invasive cancer cells, but not in non-invasive cells (Saxena et al. 2011). Chen et al. have shown that the expression of Twist1 causes resistance to an anthracycline, a class of drugs including doxorubicin and epirubicin, by regulating the expression of P-gp in the bladder cancer cell line (Chen et al. 2012).

In vivo tumorigenic assays show that Twist1-overexpressing cells, which are resistant to cisplatin, result in the widespread dissemination of tumors lining the peritoneal cavity walls (Roberts et al. 2016). Twist1 is believed to drive cisplatin resistance via upregulation of *L1CAM*, *GAS6*, and Akt signaling pathways in an ovarian cancer model (Roberts et al. 2016). Suppression of Twist1 expression by introducing miR-186 renders the ovarian cancer cells to overcome cisplatin resistance (Zhu et al. 2016).

Because the high expression level of Twist1 affects the responses to various chemotherapies in different types of cancer, the proper modulation or genetic ablation of Twist1 needs to be studied for the further possibility to restore sensitivity to the cancer drugs.

### Drug resistance: ZEB1

The ZEB transcription factor family consists of ZEB1 (also named TCF8) and ZEB2 (also known as SIPI) (Anose and Sanders 2011). ZEB family proteins contain two ZnF domains, N-terminal ZnF and C-terminal ZnF. The helix-loop-helix motif of ZEB allows for binding to the E-box within the E-cadherin promoter region with high specificity (Peinado et al. 2007). Phosphorylation within C-terminal ZnF inhibits the binding of ZEB to DNA and its transcriptional activity (Llorens et al. 2016).

ZEB1 is correlated with resistance to various drugs in many types of cancer (Witta et al. 2006; Arumugam et al. 2009; Tryndyak et al. 2010; Chang et al. 2011). MDR1 reduces the intracellular accumulation of drugs such as cisplatin, 5-FU, and doxorubicin, by functioning as an ATP-dependent drug outflow pump (Janigro et al. 2006; McCormick et al. 2015). Yoshida et al. demonstrated that ZEB1 overexpression acquires the resistance of EGFR-TKI in human NSCLC samples and cells (Yoshida et al. 2016).

Pancreatic cancer cells show different sensitivity to multiple chemotherapeutic drugs, such as gemcitabine, 5-FU, and cisplatin; MIAPaCa-2, PANC-1, Hs766T, AsPC-1, and Mpanc96 cell lines are resistant, whereas CFPAC-1, L3.6pl, BxPC-3, and SU86.86 are sensitive to the drugs (Arumugam et al. 2009). The two distinct groups of cell lines differ in the expression pattern of EMT-related genes, mainly ZEB1. Knockdown of ZEB1 restores drug sensitivity and increases the expression of epithelial markers in cancer cells showing mesenchymal characteristics (Arumugam et al. 2009).

A colon cancer cell line with oxaliplatin (OXA) resistance, HCT116/OXA, shows a high expression level of ZEB1 along with mesenchymal markers, including vimentin, MMP-2, and MMP-9 (Guo et al. 2017). Silencing of ZEB1 restores sensitivity to OXA in HCT116/OXA, and the inverse correlation between ZEB1 expression level and OXA sensitivity occurs both in vitro and in vivo (Guo et al. 2017). In addition, ZEB1 expression level is higher in histone deacetylase inhibitor (HDACi) butyrate-resistant colorectal cancer cells (Lazarova and Bordonaro 2017). These cancer cells lack the expression of histone acetyltransferase p300, which associates with β-catenin and mediates the transcriptional activity of the Wnt signaling pathway (Lazarova and Bordonaro 2017). Interestingly, mutations in the Wnt signaling pathway are a major initiating event in colorectal cancer and regulate ZEB1 expression (Sanchez-Tillo et al. 2013). Therefore, ZEB1 expression is correlated with both cancer aggressiveness and with the responses of cancer to butyrate.

Axl as a receptor tyrosine kinase is activated by vitamin K‐dependent protein, namely growth arrest-specific protein 6 (GAS6) (Zhu et al. 2019). GAS6/Axl signaling pathway drives the survival, proliferation and invasion of cancer cells (Zhu et al. 2019). Moreover, Axl overexpression has been reported to be associated with poor prognosis in a wide range of cancers and to play an essential role in metastasis (Dunne et al. 2014; Lee et al. 2014; Reichl et al. 2015; Brand et al. 2015; Hattori et al. 2016). According to the study, Axl has a correlation with the downstream targets of breast cancer specimens, and breast cancer prognosis is not good (Wang et al. 2016). Wang et al. showed that the Akt/GSK-3β/β-catenin cascade induced transcription of ZEB1, which resulted in doxorubicin resistance.

Ataxia-telangiectasia mutated (ATM) kinase plays a key role in the homologous recombination repair of damaged DNA (So et al. 2009). Zhang et al. reported that ZEB1 induces resistance to epirubicin with increasing expression of ATM kinase (Zhang et al. 2018). They also found that the expression of ZEB1 in chemo-resistant breast cancer is significantly higher than in chemo-sensitive breast cancer (Zhang et al. 2018). Interestingly, their report shows that increased expression of ATM kinase by ZEB1 enhances DNA repair in response to the epirubicin-induced DNA breakage and thus confers resistance to epirubicin (Zhang et al. 2018).

The other resistant mechanism related to ZEB1 includes long non-coding RNA of ZEB1, ZEB1-antisense 1 (ZEB1-AS1). ZEB1-AS1 is expressed from the promoter region of *ZEB1* and regulates ZEB1 expression positively (Su et al. 2017). The dysregulation of ZEB1-AS1 plays a pivotal function in tumorigenesis and tumor development (Zhao et al. 2019). Li et al. have shown that ZEB1-AS1 is upregulated in hepatocellular carcinoma and breast cancer cells and promotes metastasis (Li et al. 2016). ZEB1-AS1 regulates ZEB1 expression by binding to miR-129-5p competitively. Silenced ZEB1-AS1 inhibits resistance to cisplatin and promotes apoptosis in MCF-7 cells via upregulation of miR-129-5p and downregulation of ZEB1, Bcl-2, MDR1, and P-gp (Gao et al. 2020). The implication that ZEB1 plays a critical role in drug resistance is growing as more cases are proposed. For restoring sensitivity to cancer therapies, the appropriate approaches regulating EMT-TFs, including Snail, Twist1, and ZEB1, are required. The following section describes several strategies targeting these EMT-TFs.

### Targeting EMT-TFs to suppress cancer development and overcome drug resistance

Because the EMT process and its transcription factors drive drug resistance, the inhibitors of the process and factors will be beneficial for chemotherapy or targeted therapy (Du and Shim 2016).

In mutant K-RAS-driven cancer cells, Snail binds to the DNA-binding domain of p53 and prevents p53 from DNA binding and further apoptosis or senescence induced by p53 activity (Lee et al. 2009). Although a few inhibitors are developed to bind and inhibit Snail directly, GN25 and GN29 bind Snail and inhibit this interaction between Snail and p53 (Lee et al. 2010). The K-RAS mutated-cancer cells treated with the compounds show reduced proliferation and tumor progression. Unfortunately, these compounds have a limitation in that they are only effective in K-RAS-driven cancer cells harboring wild-type p53.

Recently, Hong et al. identified a small-molecule CYD19 that binds Snail with a high affinity and inhibits the interaction of Snail with CREB-binding protein (CBP)/p300 (Li and Balazsi 2018). The disrupted interaction leads to impairment of CBP/p300-mediated Snail acetylation and accelerated degradation of Snail via the proteasomal pathway. Snail-dependent EMT, cancer stem cell expansion, and metastasis are inhibited in vivo as Snail degradation is induced by CYD19.

Given that Snail binds to the consensus E-box sequence, Vistain et al. utilized an amine-modified E-box oligonucleotide conjugated to the cobalt(III) complex to generate Co^III^-Ebox (Vistain et al. 2015). As Co^III^-E-box is introduced into the cells, Snail binds to Co^III^-E-box, and the endogenous E-cadherin gene expression is no longer repressed by Snail. Co^III^-E-box blocks the binding of Snail to the promoter regions of its target genes, thus further inhibiting EMT in breast cancer cells.

A few direct inhibitors of Snail target the interaction of Snail with its binding partners as described above. The other approach to inhibit Snail is to modulate the regulatory mechanisms of Snail. For instance, the stability of Snail can be enhanced by Dub3, a deubiquitinase of Snail. Dub3-mediated Snail stabilization is disrupted by a Dub3-specific inhibitor, WP1130, and Snail-driven metastasis is inhibited in breast cancer (Wu et al. 2017).

Harmine, a harmala alkaloid, is as a novel Twist1 inhibitor that successfully inhibits cell dissemination, growth of invadopodia in 3D culture, and proliferation of NSCLC cells (Yochum et al. 2017; Zhang et al. 2020). Harmine induces the degradation of Twist1 heterodimerized with E2A protein, a dimer partner of Twist1 (Yochum et al. 2017). In addition, harmine shows anti-cancer activity via Twist1 suppression in K-RAS mutant NSCLC mouse models (Yochum et al. 2017).

ABT-263 is an orally available analog that binds and inhibits anti-apoptotic Bcl-2/Bcl-X_L_. Furthermore, ZEB1 suppresses transcription of pro-apoptotic *BCL2L11*, which results in resistance to ABT-263. However, FK228 (also romidepsin), known as HDACi, attenuated ZEB1-induced ABT263 resistance by up-regulating Bcl-2-like protein 11 expression (Inoue-Yamauchi and Oda 2020).

The underlying mechanism of EMT-related resistance to doxorubicin is also highly dependent on miRNA expression and activity. A study by Chen et al. shows that the expression of miR-200 is reduced significantly when MCF-7 cells become resistant to doxorubicin (Chen et al. 2013). This miRNA targets the mRNA of ZEB1/2 directly (Park et al. 2008). Ectopic expression of miR-200 induces E-cadherin upregulation, vimentin downregulation, and decreased motility.

## Concluding remarks

Snail, Twist, and ZEB families not only induce EMT but also are correlated with drug resistance, all of which makes cancer therapy more challenging. We have described the cancer drug resistance regarding EMT-TFs and targeting strategies of EMT-TFs. Although the strategies targeting the EMT-TFs directly are not in the clinical trials yet, the relevance of the EMT-TFs in metastatic cancer is significant in vitro, in vivo, and in clinical data. Investigation into the regulatory mechanisms of the EMT-TFs in correlation with cancer therapeutic resistance will develop novel targeting strategies towards the EMT-TFs. Eventually, different approaches to understanding the function and regulation of EMT-TFs will shed light on metastatic cancer therapy.
